# Nomogram prediction for the prediction of clinical pregnancy in Freeze-thawed Embryo Transfer

**DOI:** 10.1186/s12884-022-04958-8

**Published:** 2022-08-08

**Authors:** Qian Zhang, Xiaolong Wang, Yuming Zhang, Haiou Lu, Yuexin Yu

**Affiliations:** 1 Department of Reproductive Medicine, General Hospital of Northern Theater Command, Shenhe District, No. 83, Wenhua Road, Shenyang, 110016 China; 2 Department of Forensic Pathology, School of Forensic Medicine, China Medical University, Shenyang, 110122 China

**Keywords:** Clinical pregnancy outcome, Nomogram, Endometrial receptivity, Frozen-thawed embryo transfer

## Abstract

**Background:**

This study aimed to identify multiple endometrial receptivity related factors by applying non-invasive, repeatable multimodal ultrasound methods. Combined with basic clinical data, we further established a practical prediction model for early clinical outcomes in Freeze-thawed Embryo Transfer (FET).

**Methods:**

Retrospective analysis of clinical data of infertility patients undergoing FET cycle in our Center from January 2017 to September 2019. Receiver operating characteristic (ROC) curve and decision curve analyses were performed by 500 bootstrap resamplings to assess the determination and clinical value of the nomogram, respectively.

**Results:**

A total of 2457 FET cycles were included. We developed simple nomograms that predict the early clinical outcomes in FET cycles by using the parameters of age, BMI, type and number of embryos transferred, endometrial thickness, FI, RI, PI and number of endometrial and sub-endometrial blood flow. In the training cohort, the area under the ROC curve (AUC) showed statistical accuracy (AUC = 0.698), and similar results were shown in the subsequent validation cohort (AUC = 0.699). Decision curve analysis demonstrated the clinical value of this nomogram.

**Conclusions:**

Our nomogram can predict clinical outcomes and it can be used as a simple, affordable and widely implementable tool to provide guidance and treatment recommendations for FET patients.

**Supplementary Information:**

The online version contains supplementary material available at 10.1186/s12884-022-04958-8.

## Background

Infertility occurs in more than 15% of married couples and has a significant impact on patients and families [1]. In vitro fertilization and embryo transfer (IVF-ET) is now widely used in the treatment of infertility, and the number of cycles performed in IVF is increasing dramatically worldwide. The number and proportion of FET cycles is also steadily increasing [2]. Successfully predicting the probability of pregnancy in the IVF cycle is a long-standing problem [3]. It is important to correctly predict pregnancy, both from a clinician's patient perspective [3, 4] and from an economic perspective [5]. Through the predictive model, clinicians can estimate the probability of successful pregnancy according to the patient's own situation, better carry out IVF-ET consultation to make the optimal clinical decision and patient selection, and patients will therefore have a certain psychological expectation of the treatment outcome of this cycle.

The success of the assisted reproductive technology cycle depends mainly on age, embryo quality and endometrial receptivity [6]. As we know, endometrial receptivity refers to a state in which the endometrium allows the embryo to locate, adhere and implant [7]. The synchronization of endometrium and embryo development is a requirement for embryo implantation. With the improvement and maturity of laboratory technology, most couples can obtain high-quality embryos. Therefore, the endometrial receptivity is very important for judging the patient's expected treatment outcome. The application of endometrial receptivity has important guiding significance for treatment which can effectively improve the success rate of IVF, but one of the main problems is the lack of actual clinical trials to evaluate endometrial receptivity.

More recently, microscopy, flow cytometry and molecular advancements have allowed further understanding of the cross-talk between the embryo and the endometrium. Methods for evaluating embryo receptivity in the endometrium are also increasing, including morphology, genomics, transcriptomics, proteomics, metabolomics, etc. [8]. Morphological indicators are still commonly used in clinical evaluation. Ultrasound testing is still the most widely used non-invasive evaluation method in most reproductive medical institutions because of its convenience, non-invasiveness and repeatability. Ultrasound evaluation of endometrial receptivity can detect a variety of imaging features and provide a variety of feature parameter combinations. Most previous pregnancy prediction models are constructed based on clinical data and rarely consider the intrauterine receptivity on transplantation date. Although ultrasound assessment has been used and shown to be helpful in predicting outcomes in assisted reproduction patients, but the problem was the absence of a consensus on which performance measures to use in forecasting models and how to interpret them. The combination of multiple ultrasound imaging parameters and clinical data as the best method for prediction remains to be developed. Combining a variety of ultrasound imaging parameters and clinical data as the best method for prediction remains to be developed.

In our study, we used a series of multimodal ultrasound methods to assess endometrial receptivity related to uterus condition, in order to achieve complementary advantages and better evaluate the optimal period of endometrial implantation. Through multi-dimensional comprehensive evaluation, an individualized ultrasonic standard for endometrial receptivity evaluation was sought to predict the pregnancy outcome of Freeze-thawed Embryo Transfer more quickly and accurately.

## Materials and methods

### Study design and patients

**Fig. 1:**
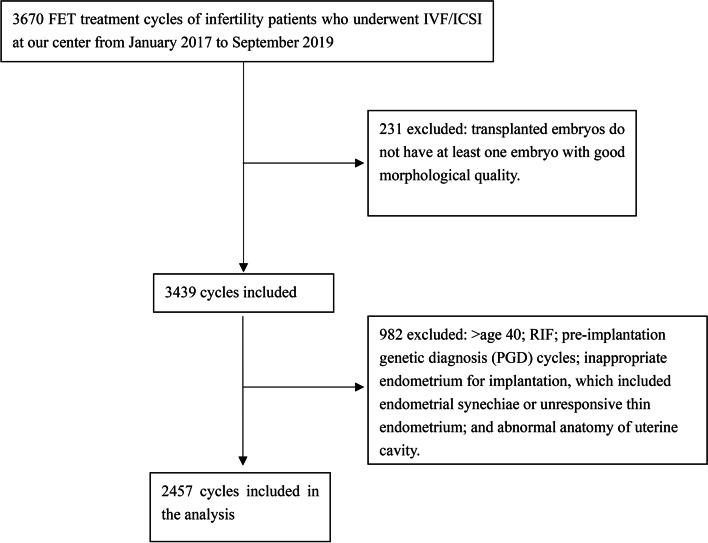
Flowchart of patient screening

A retrospective cohort analysis was performed using data from FET treatment cycles of infertility patients who underwent IVF/ICSI at our center from January 2017 to September 2019. General demographic characteristics were investigated, including infertility history, relevant clinical and laboratory data, and treatment outcomes. Inclusion criteria were women younger than 40 years who had at least one embryo with good quality in morphology. Exclusion criteria were: pre-implantation genetic diagnosis cycles, recurrent implantation failure (RIF), inappropriate endometrium for implantation, which included endometrial synechiae or unresponsive thin endometrium and abnormal anatomy of uterine cavity. A flow diagram is showed in Fig. 1. A total of 2457 FET cycles were analyzed. All patients’ information were numbered, and the computer randomly selected numbers at a ratio of 3:1 to form a training cohort (*n* = 1853) and a validation cohort (*n* = 604).

This was a retrospective study of routinely collected clinical data, and exemption from informed consent was approved by the medical ethics committee of General Hospital of Northern Theater Command (registration number: 202H2019PJ003). All experimental protocols were approved by the medical ethics committee of General Hospital of Northern Theater Command and all methods were carried out in accordance with the Declarations of Helsinki.

### Data collection

Patient demographics and characteristics were collected, including female age, body mass index (BMI), infertile history, and basal levels of sex steroid hormones. The parameters related to FET cycles, included FET protocols, number and type of embryos transferred, the endometrial thickness and endometrial pattern on day of transplantation and other ultrasound measurement indicators related to endometrial and sub-endometrial blood flow. Among them, these blood flow indicators were: vascularization index (VI), flow index (FI), resistive index (RI), pulsatility index (PI), blood flow type and number of blood flow branches of spiral artery. Three grades were considered according to the number of blood flow branches: (A) ≤ 5, (B) ≥ 9, and (C) between 5–9. The outcomes measured were clinical pregnancy rate per embryo transfer cycle.

### Treatment protocol

FET protocols in our center were mainly divided into natural cycle after spontaneous ovulation and artificial cycles (hormone replacement treatment cycles) based on the regularity of the menstrual cycle. For the natural cycles, the assessment of endometrial thickness, follicle growth, and ovulation by transvaginal ultrasound examination were initiated from cycle days 8 to 10 by the same physician. When the diameter of the dominant follicle was between 16 to 20 mm, hormone levels should be measured. The date of ovulation was determined by monitoring the levels of serum estradiol, progesterone and luteinizing hormone, combined with transvaginal ultrasound to identify follicular rupture. Thawing and transferring of embryo was planned 3–5 days after ovulation. For artificial cycles, administration of oral estradiol (Progynova; Bayer; Leverkusen, Germany) was initial with 4 mg/day from cycle day 3. This dose was adjusted based on endometrial thickness every four days. After 12–14 days, an ultrasound was performed and a serum progesterone level was determined. When the endometrial thickness reached 6.0 mm, intramuscular administration of progesterone in oil (40 mg) and oral progesterone (dydrogesterone 20 mg) were provided and maintained until embryo transferred. After embryo transfer, the luteum support protocol was the same as before.

### Ultrasound measurement

All the ultrasound measurement assessments were carried out by specialist sonographers using the same standardized protocols on the same ultrasound machines in our department (GE Voluson E8, the United States).

Endometrial thickness and pattern were measured as described in our previous paper [9]. Endometrial spiral arterial blood flow detects blood flow signals under and around the intima. The power Doppler blood flow imaging function was activated, and the sensitive state was adjusted to observe the branching of endometrial blood flow. The median sagittal section of the uterus, the region of interest surrounding the intima and 1/3 of the myometrium at the endometrium, and the activation of Doppler flow imaging. The pulse repetition frequency PRF was set at 0.6 MHz. The number of endometrial and subendometrial blood flow branches was observed and recorded.

The waveforms from the spiral artery were obtained by placing the Doppler over the color area and activating the pulsed Doppler function. After at least 5 consecutive waveforms were obtained, the resistive index (RI) and pulsatility index (PI) were checked. To reduce errors in blood flow measurements, the RI and PI were measured in 3 different spiral arteries in each phase, and the mean value was used in the analysis. The endometrial-subendometrial blood flow distribution pattern was determined by demonstrating transvaginal power Doppler flow mapping in the subendometrial and endometrial regions. The distribution pattern was based on Applebaums criteria [10], summarized as follows: I, vessels penetrating the outer hypoechogenic area surrounding the endometrium but not entering the hyperechogenic outer margin; II, vessels penetrating the hyperechogenic outer margin of the endometrium but not entering the hypoechogenic inner area; III, vessels entering the hypoechogenic inner area. The 3D power Doppler indices below the endometrium were measured using the automated VOCALTM for the 3D power Doppler histogram analysis. VI was defined as the ratio between color and all voxels number which represents the endometrial vascularity and expressed as a percentage of the endometrial volume. FI was defined as the mean power Doppler signal intensity within the endometrium which represents the mean flow intensity.

All FET patients underwent transvaginal ultrasonic examination in the morning on the embryo transfer day.

### Embryo transfer and embryo score

Prior to embryo transfer, embryos were graded according to their developmental speed, fragmentation degree and evenness of cleavage sphere. The cleavage embryos with 7 ~ 9 blastomere, uniform cytoplasm, regular morphology, fragmentation < 10% were considered as high-quality embryos. For blastocysts, the Gardner grading method [11] was used to score blastocysts, and ≥ 3BB and above were considered as high-quality embryos.

### Pregnancy

Serum human chorionic gonadotropin (HCG) tests was performed 14 days after embryo transfer. If it was positive, ultrasound examination was performed 10–14 days later to confirm intrauterine pregnancy. Clinical pregnancy was defined as one in which a positive pregnancy was accompanied later by ultrasonographic evidence of an intrauterine gestational sac.

### Statistical analysis

Characteristics of all participants stratified by training/validation cohort were presented as means (standard deviations) or medians (interquartile ranges) for continuous variables, and as frequencies (percentage) for categorical variables. And categorical variables were presented as percentages. COX univariate and multivariate survival analysis were applied to evaluate the impact of basic clinical data and easily accessible ultrasound measurement indicators to clinical outcomes in FET cycles. Then, in univariate analysis, clinical outcomes related variables (*P* < 0.05) were included in Stepwise Akaike Information Criterion (step AIC) analysis to select out factors for the establishment of a probability predictive nomogram. C-index and decision curve analysis (DCA) [12] were performed to determine the clinical useful-ness of the model and weighed to obtain a net benefit of making a decision. C-index of discrimination and calibration curves were presented to qualify the predictive accuracy of the nomogram. And 500 bootstrap re-samplings were performed to validate this model.At last, we also established a full model and a multivariable fractional polynomial (MFP) model; predictive accuracy is presented in Supplemental Appendix (Table S1). In addition, we conducted ROC analyses to determine the optimal cutoff values of each risk factor (Table 5). The ROC curves of each risk factor are presented in Supplemental Appendix (Fig. S1).

All the statistical analyses were performed with statistical packages R (http://www.R-project.org) and EmpowerStats (www.empowerstats.com, X&Y Solutions, Inc., Boston, MA).

## Results

### Baseline characteristics

A total of 1853 and 604 FET cycles were included in the final analysis as the training and validation cohorts, respectively. The clinical pregnancy rates were approximately 62.98% and 62.09% in the training and validation cohorts, respectively. The difference between the two sets was insignificant (Table 1). For the training cohort, Table 2 displays the baseline characteristics grouped as those with or without incident clinical pregnancy. The female age, level of baseline FSH, RI of spiral artery were lower with significant differences in the pregnancy group. The level of AMH, proportion of blastocyst, endometrial thickness on transplantation day, VI, FI and the number of blood flow branches of endometrial and sub-endometrial blood were higher with significant differences in the pregnancy group. The level of Endometrial blood flow classification is also higher. Univariate logistic regression analysis also reached similar conclusions (Table 3). In the multivariate logistic analyses, on the basis of the odds ratio (95% CI) and *P* value results, Female age, BMI, type of embryos transferred, No. of embryos transferred, endometrial thickness, No. of sub-endometrial blood, FI, RI, and PI were significantly correlated with pregnancy outcome (Table 3).

**Table 1:** Characteristics of the training and validation cohorts(*N* = 2457)

Characteristic	Training cohort (*n* = 1853)	Validation cohort (*n* = 604)	*P*-value
Female age (years)	32.50 ± 3.60	32.62 ± 3.74	0.412
BMI (kg/m^2^)	23.44 ± 3.78	23.57 ± 3.82	0.621
AMH (ng/ml)	3.99 ± 3.10	4.19 ± 3.32	0.362
Baseline FSH (IU/L)	6.11 ± 2.30	6.05 ± 2.46	0.586
Duration of infertility (years)	4.07 ± 3.07	4.34 ± 3.34	0.202
Type of infertility, n(%)			0.722
Primary infertility	991 (53.48%)	318 (52.65%)	
Secondary infertility	862 (46.52%)	286 (47.35%)	
Infertility diagnosis, n(%)			0.696
Female factor	1104 (59.58%)	370 (61.26%)	
Male factor	226 (12.20%)	70 (11.59%)	
Multiple factors	461 (24.88%)	149 (24.67%)	
Unexplained and other	62 (3.35%)	15 (2.48%)	
FET protocols, n(%)			0.212
Artificial cycle	225 (12.14%)	62 (10.26%)	
Natural cycle	1628 (87.86%)	542 (89.74%)	
Type of embryos transferred, n(%)			0.576
Cleavage embryo	1009 (54.45%)	321 (53.15%)	
Blastocyst	844 (45.55%)	283 (46.85%)	
No. of embryos transferred, n(%)			0.847
1	511 (27.58%)	169 (27.98%)	
2	1342 (72.42%)	435 (72.02%)	
Endometrial thickness (cm)	1.00 ± 0.22	0.99 ± 0.20	0.994
Endometrial pattern, n (%)			-
Type A	639 (34.48%)	190 (31.46%)	
Type non-A	1214 (65.52%)	414 (68.54%)	
VI	5.68 ± 3.67	5.56 ± 3.51	0.621
FI	32.96 ± 5.08	32.87 ± 5.15	0.734
RI	0.51 ± 0.09	0.51 ± 0.09	0.672
PI	0.75 ± 0.21	0.75 ± 0.20	0.981
No. of Sub-endometrial blood, n (%)			0.797
A	87 (4.70%)	31 (5.13%)	
B	1076 (58.07%)	356 (58.94%)	
C	690 (37.24%)	217 (35.93%)	
Endometrial blood flow classification, n (%)			0.486
I	136 (7.34%)	53 (8.77%)	
II	1660 (89.58%)	531 (87.91%)	
III	57 (3.08%)	20 (3.31%)	
Pregnancy outcomes			0.693
Non-pregnancy	686 (37.02%)	229 (37.91%)	
Pregnancy	1167 (62.98%)	375 (62.09%	

Data are shown as means ± SD, median (interquartile range), or no. (%)

*BMI* Body mass index, *AMH* AntiMullerian hormone, *FSH* follicular stimulating hormone, *VI* Vascularization index, *FI* Flow index, *RI* Resistive index, *PI* Pulsatility index

**Table 2:** Baseline characteristics according to the pregnancy outcomes in the training cohort (*N* = 1853)

Characteristic	Non-pregnancy (*n* = 686)	Pregnancy (*n* = 1167)	*P*-value
Female age (years)	33.06 ± 3.66	32.17 ± 3.53	< 0.001
BMI (kg/m^2^)	23.23 ± 3.64	23.57 ± 3.86	0.063
Duration of infertility (years)	4.22 ± 3.24	3.99 ± 2.96	0.110
AMH (ng/ml)	3.68 ± 3.08	4.17 ± 3.10	0.001
Baseline FSH (IU/L)	6.31 ± 2.29	5.99 ± 2.30	0.004
Endometrial thickness (cm)	0.96 ± 0.22	1.02 ± 0.21	< 0.001
VI	5.00 ± 3.17	6.08 ± 3.88	< 0.001
FI	32.20 ± 6.28	33.41 ± 4.15	< 0.001
RI	0.52 ± 0.11	0.50 ± 0.08	< 0.001
PI	0.76 ± 0.23	0.75 ± 0.20	0.100
FET protocols, n(%)			0.129
Artificial cycle	73 (10.64%)	152 (13.02%)	
Natural cycle	613 (89.36%)	1015 (86.98%)	
Type of infertility, n(%)			0.069
Primary infertility	348 (50.73%)	643 (55.10%)	
Secondary infertility	338 (49.27%)	524 (44.90%)	
Infertility diagnosis, n(%)			0.230
Female factor	422 (61.52%)	682 (58.44%)	
Male factor	70 (10.20%)	156 (13.37%)	
Multiple factors	170 (24.78%)	291 (24.94%)	
Unexplained and other	24 (3.50%)	38 (3.26%)	
Type of embryos transferred, n(%)			< 0.001
Cleavage embryo	430 (62.68%)	579 (49.61%)	
Blastocyst	256 (37.32%)	588 (50.39%)	
No. of embryos transferred, n(%)			0.929
1	190 (27.70%)	321 (27.51%)	
2	496 (72.30%)	846 (72.49%)	
Endometrial pattern, n (%)			0.718
Type A	233 (33.97%)	406 (34.79%)	
Type non-A	453 (66.03%)	761 (65.21%)	
No. of Sub-endometrial blood, n (%)			< 0.001
A	69 (10.06%)	18 (1.54%)	
B	455 (66.33%)	621 (53.21%)	
C	162 (23.62%)	528 (45.24%)	
Endometrial blood flow classification, n (%)			< 0.001
I	65 (9.48%)	71 (6.08%)	
II	611 (89.07%)	1049 (89.89%)	
III	10 (1.46%)	47 (4.03%)	

Data are shown as means ± SD, median (interquartile range), or no. (%)

*BMI* Body mass index, *AMH* AntiMullerian hormone, *FSH* Follicular stimulating hormone, *VI* Vascularization index, *FI* Flow index, *RI* Resistive index, *PI* Pulsatility index

**Table 3:** The univariate and multivariate logistic regression analysis for factors associated pregnancy outcomes in the training cohort (*N* = 1853)

Characteristic	**Univariable**		**Multivariable**	
	OR (95% CI)	*P* value	OR (95% CI)	*P* value
Female age (years)	0.93 (0.91, 0.96)	< 0.0001	0.96 (0.93, 0.99)	0.0221
FET protocols, n(%)				
Artificial cycle	1.0	-	1.0	-
Natural cycle	0.80 (0.59, 1.07)	0.1299	0.84 (0.61, 1.16)	0.2975
BMI (kg/m^2^)	1.02 (1.00, 1.05)	0.0637	1.04 (1.01, 1.07)	0.0046
Type of infertility, n(%)				
Primary infertility	1.0	-	1.0	-
Secondary infertility	0.84 (0.69, 1.01)	0.0687	0.94 (0.75, 1.18)	0.5851
Duration of infertility (years)	0.98 (0.95, 1.01)	0.1104	0.99 (0.95, 1.02)	0.4431
Infertility diagnosis, n(%)				
Female factor	1.0	-	1.0	-
Male factor	1.38 (1.01, 1.87)	0.0402	1.39 (0.99, 1.94)	0.0542
Multiple factors	1.06 (0.85, 1.33)	0.6161	1.14 (0.89, 1.46)	0.2993
Unexplained and other	0.98 (0.58, 1.66)	0.9390	1.05 (0.59, 1.85)	0.8742
AMH (ng/ml)	1.05 (1.02, 1.09)	0.0012	1.02 (0.98, 1.06)	0.3020
Baseline FSH (IU/L)	0.94 (0.90, 0.98)	0.0048	1.00 (0.95, 1.05)	0.9309
Type of embryos transferred, n(%)				
Cleavage embryo	1.0	-	1.0	-
Blastocyst	1.71 (1.41, 2.07)	< 0.0001	1.95 (1.50, 2.55)	< 0.0001
No. of embryos transferred, n(%)				
1	1.0	-	1.0	-
2	1.01 (0.82, 1.25)	0.9294	1.63 (1.22, 2.18)	0.0008
Endometrial thickness (cm)	3.38 (2.14, 5.33)	< 0.0001	2.61 (1.57, 4.35)	0.0002
Endometrial pattern, n (%)				
Type A	1.0	-	1.0	-
Type non-A	0.96 (0.79, 1.18)	0.7183	1.00 (0.80, 1.24)	0.9695
No. of Sub-endometrial blood, n (%)				
A	1.0	-	1.0	-
B	5.23 (3.07, 8.91)	< 0.0001	4.276(2.42, 7.50)	< 0.0001
C	12.49 (7.22, 21.61)	< 0.0001	8.55 (4.65, 15.73)	< 0.0001
VI	1.09 (1.06, 1.13)	< 0.0001	1.02 (0.99, 1.06)	0.1959
FI	1.05 (1.03, 1.07)	< 0.0001	1.04 (1.01, 1.06)	0.0010
Endometrial blood flow classification, n (%)				
I	1.0	-	1.0	-
II	1.57 (1.11, 2.23)	0.0116	0.78 (0.52, 1.16)	0.2132
III	4.30 (2.01, 9.21)	0.0002	1.95 (0.85, 4.46)	0.1146
RI	0.08 (0.03, 0.25)	< 0.0001	0.01 (0.00, 0.08)	< 0.0001
PI	0.69 (0.44, 1.08)	0.1024	3.98 (1.42, 11.14)	0.0087

Data are as odds ratio (95% CI), *P* value

Data are shown as means ± SD, median (interquartile range), or no. (%)

*BMI* Body mass index, *AMH* AntiMullerian hormone, *FSH* Follicular stimulating hormone, *VI* Vascularization index, *FI* Flow index, *RI* Resistive index, *PI* Pulsatility index

### Establishment of a nomogram for predicting clinical outcomes in FET cycles

**Fig. 2:**
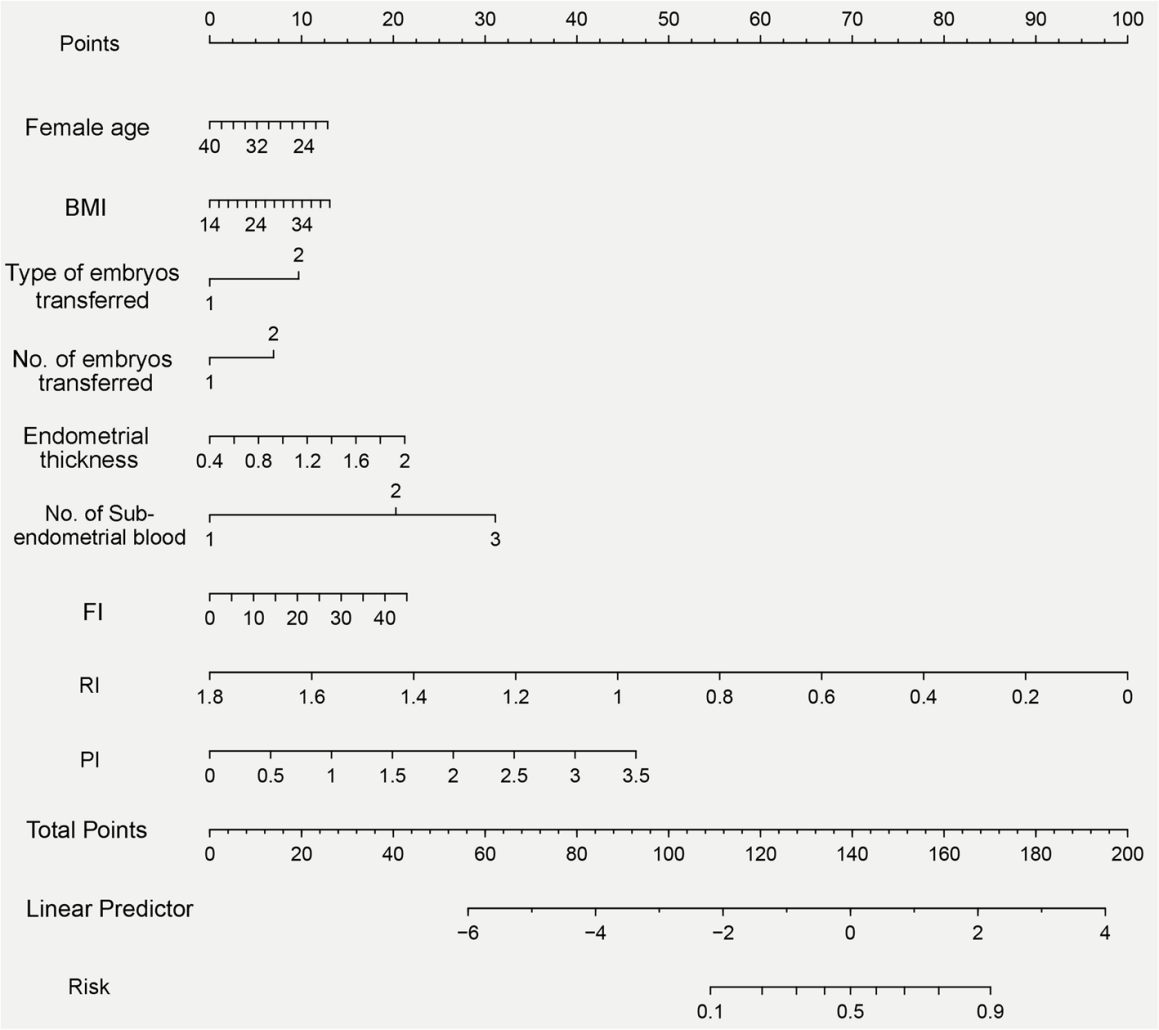
Nomogram to predict the probability of early clinical pregnancy for FET patients. To estimate an individual’s probability of clinical pregnancy for FET patients, locate her value on each variable axis. Draw a vertical line from that value to the top Points scale for determining how many points are assigned by that variable value. Then, the points from each variable value are summed. Locate the sum on the Total Points scale and vertically project it onto the bottom axis, thus obtaining a personalized probability of clinical pregnancy for FET patients. Using bootstrap resampling (times = 500)

The nomogram of the stepwise model was drawn to provide a quantitative and convenient tool in predicting clinical pregnancy outcomes by using female age, BMI, type of embryos transferred, the number of embryos transferred, endometrial thickness, the number of sub-endometrial blood, FI, RI, and PI in the training cohort (Fig. 2). To estimate an individual’s incidence of clinical pregnancy, her value is located on each variable axis. A vertical line is drawn from that value to the top Points scale for determining how many points are assigned by that variable value. Then, the points from each variable value are summed. The sum is located at the Total Points scale and is vertically projected onto the bottom axis, thus obtaining a personalized incidence of clinical pregnancy.

**Fig. 3:**
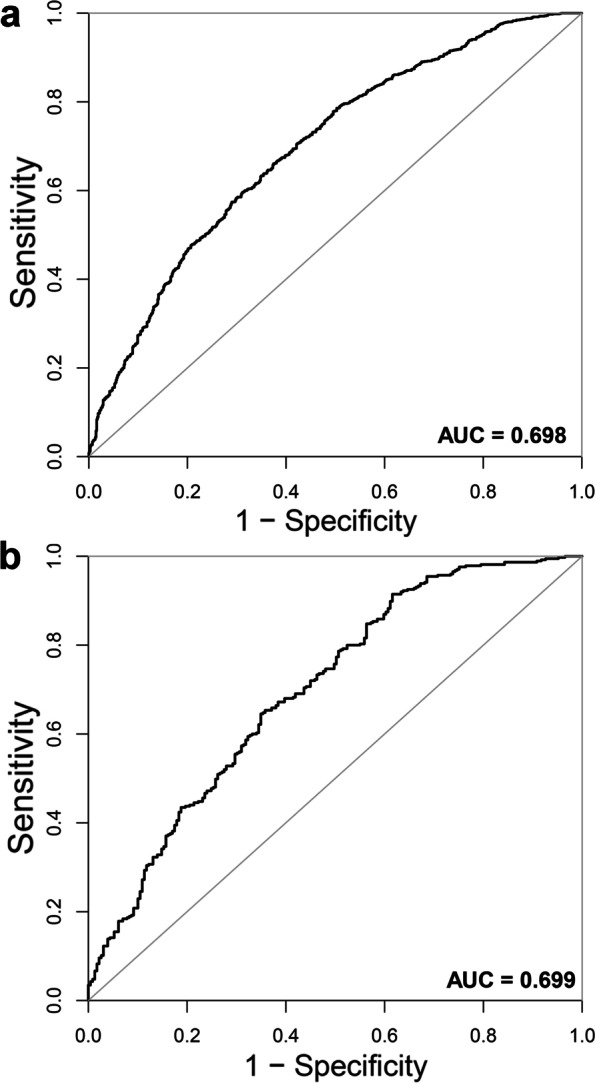
The ROC curves of the nomogram for probability of early clinical pregnancy for FET patients in the training cohort and validation cohort. **a** In the training cohort, the AUCs was 0.698 (95% CI, 0.673–0.722), respectively. **b** In the validation cohort, the AUCs was 0.699 (95% CI, 0.656–0.743), respectively. ROC: receiver operating characteristics curves, AUC: area under curve. Using bootstrap resampling (times = 500)

The prediction accuracy of the nomogram is presented in Table 4, and ROC curves are shown in Fig. 3. The resulting model was internally validated by 500 bootstrap resamplings. In the training cohort, the area under the ROC curve (AUC) was 0.698 (95% CI, 0.673–0.722), respectively. A similar result was observed in the subsequent validation cohort. The nomogram displayed AUC of 0.699 (95% CI, 0.656–0.743), respectively. The optimal cutoff value of the nomogram was 0.670 in the training cohort. In the training cohort, the sensitivity rate was 66.07%, and the specificity percentage was 62.54%, respectively. In the validation cohort, the sensitivity rate was 91.47%, and the specificity percentage was 38.43%, respectively.

**Table 4:** Prediction performance of the nomogram for estimating the clinical pregnancy outcomes^a^

	**Training cohort**	**Validation cohort**
AUC(95%CI)	0.698(0.673–0.722)	0.699(0.656–0.743)
Cutoff value	0.670	0.486
Sensitivity,%	66.07%	91.47%
Specificity,%	62.54%	38.43%
PPV,%	75.00%	70.87%
NPV,%	52.00%	73.33%
PLR	1.764	1.486
NLR	0.543	0.222

*AUC* Area under curve, *PPV* Positive predictive value, *NPV* Negative predictive value, *PLR* Positive likelihood ratio, *NLR* Negative likelihood ratio

^a^Using bootstrap resampling (times = 500)

**Fig. 4:**
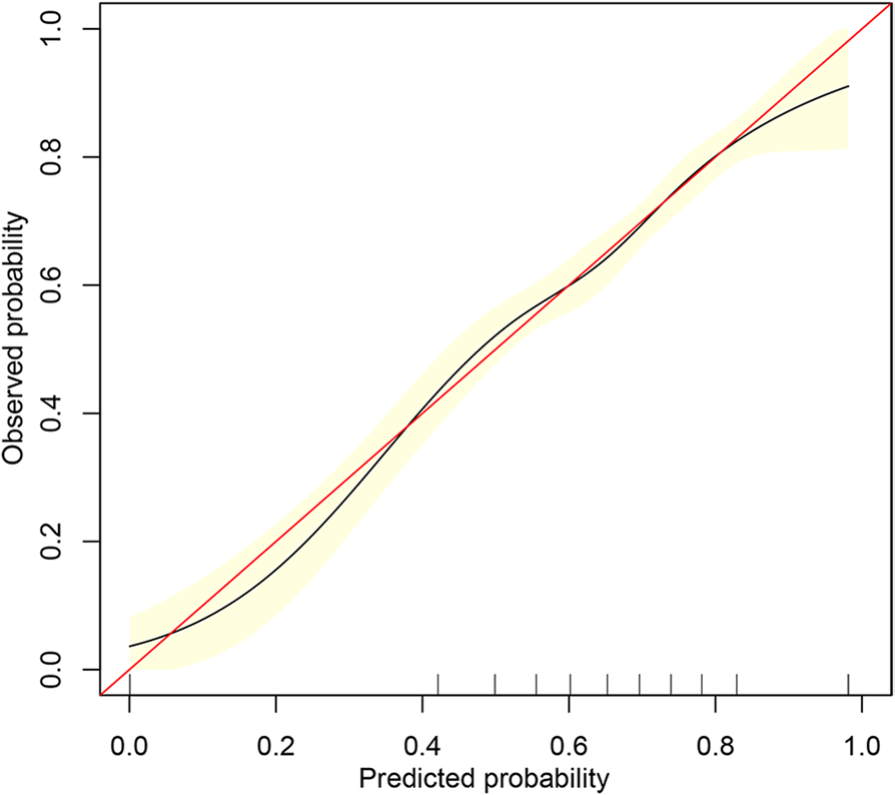
Calibration curve of the nomogram in the training cohort. The red line is reference line, and the black line is fitting. The yellow area represents the 95% CI. After 500 repetitions of bootstrap, the calibration curves showed a good correlation between the predicted probability and actual probability

The clinical pregnancy prediction accuracy was validated by the calibration curve which showed a correlation between the actual observed outcome and the prediction by the nomogram. This correlation data from the calibration curve was observed even when the nomogram prediction probability was less than 20% (Fig. 4).

**Fig. 5:**
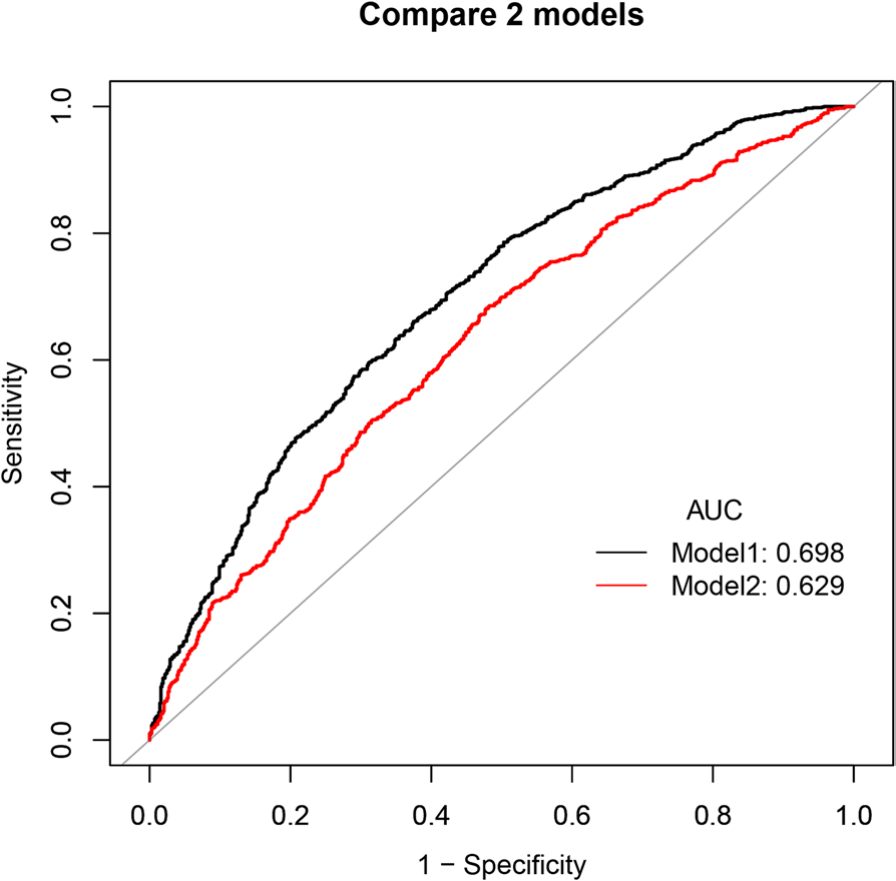
Receiver operating characteristic curves of the combined model (incorporate endometrial and sub-endometrial blood flow indicators) and model without blood flow indicators. Model 1: the combined model integrated the number of sub-endometrial blood, FI, RI, PI, age, BMI, type of embryos transferred, the number of embryos transferred and endometrial thickness. Model 2: model without blood flow indicators which only incorporated age, BMI, type of embryos transferred, the number of embryos transferred, endometrial thickness. AUC: area under curve

In addition, we compared the predictive power of nomogram integrating the number of sub-endometrial blood, FI, RI, PI, age, BMI, type of embryos transferred, the number of embryos transferred and endometrial thickness with model only incorporating age, BMI, type of embryos transferred, the number of embryos transferred and endometrial thickness by ROC curve analysis. As shown in Fig. 5, the AUC of the model combined with endometrial and sub-endometrial blood flow indicators and the without model was 0.698 and 0.629 (*P* < 0.0001), respectively, indicating that the combined model had a better performance than the model without blood flow indicators. In summary, the nomogram demonstrated fair predictive accuracy in estimating the incidence of clinical pregnancy in FET cycles.

### Decision curves for the pregnancy-predicting nomogram in FET cycles

**Fig. 6:**
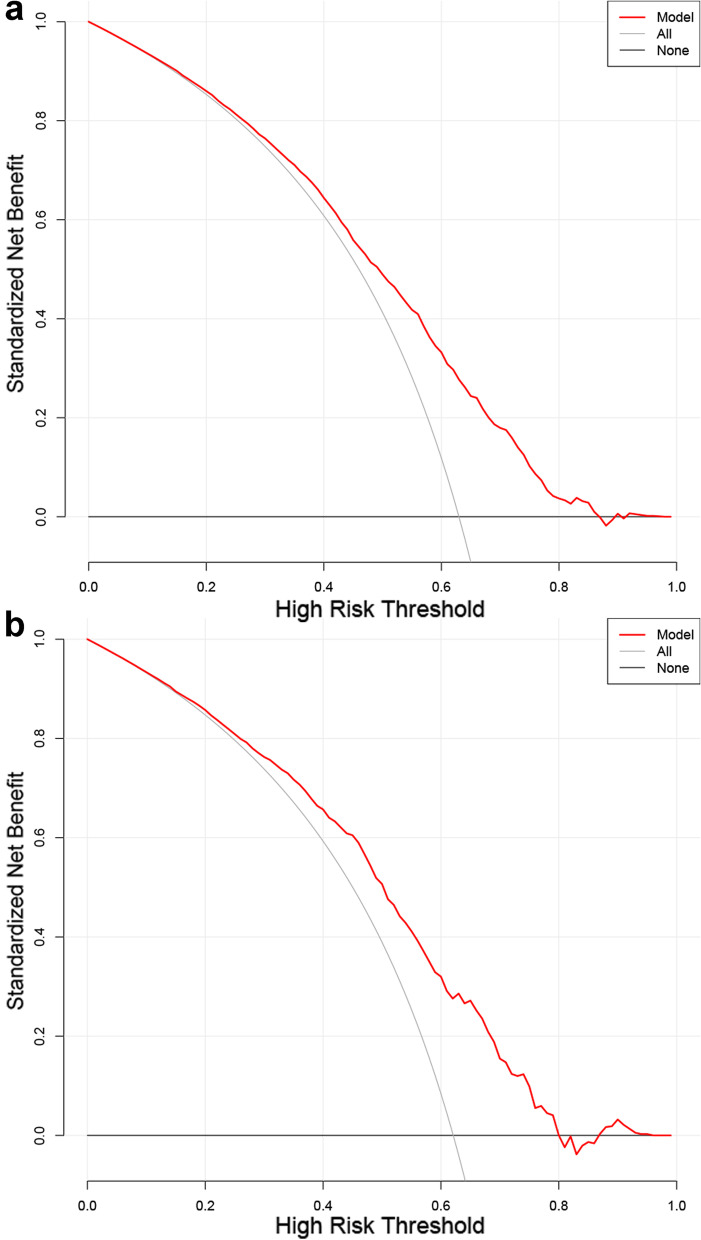
The decision curve analysis of the nomogram for probability of early clinical pregnancy in the training cohort and validation cohort. The solid bold line represents the net benefit when no participant was considered to get early clinical pregnancy, while the solid thin line represents the net benefit when all participants were considered to get early clinical pregnancy. The area among the model curve, “treat none line” (solid bold line) and “treat all line” (solid thin line), represents the clinical usefulness of the model. The farther the model curve is to the solid bold and solid thin lines, the better clinical value the nomogram holds. Using bootstrap resampling (times = 500)

Figure 6 illustrates the decision curves for the training and validation cohorts to predict the incidence of clinical pregnancy. The solid bold line represents the net benefit when no participant was considered to get early clinical pregnancy, while the solid thin line represents the net benefit when all participants were considered to get early clinical pregnancy. The area among the model curve, “treat none line” (solid bold line) and “treat all line” (solid thin line), represents the clinical usefulness of the model. The farther the model curve is to the solid bold and solid thin lines, the better clinical value the nomogram holds.

### Optimal cutoff values of related factors for clinical pregnancy prediction in FET

The optimal cutoff values of each related factor that was determined using the ROC analyses are summarized in Table 5. The cutoff values of female age, BMI, AMH, baseline FSH, endometrial thickness, VI, FI, RI, and PI were 35.5 years, 23.6 kg/ m^2^, 2.23 ng/mL, 6.49 IU/L, 0.99 mm, 4.26, 29.34, 0.53 and 0.61 respectively, to predict the clinical pregnancy in FET optimally.

**Table 5:** Optimal cutoff values of related factors for clinical pregnancy prediction ^a^

characteristic	Cutoff value	AUC	Sensitivity(%)	Specificity(%)
Female age (years)	35.50	0.567	0.813	0.282
BMI (kg/m^2^)	23.60	0.528	0.460	0.589
AMH (ng/ml)	2.23	0.571	0.723	0.389
Baseline FSH (IU/L)	6.49	0.538	0.675	0.397
Endometrial thickness (cm)	0.99	0.576	0.578	0.567
VI	4.26	0.592	0.628	0.491
FI	29.34	0.559	0.866	0.206
RI	0.53	0.607	0.540	0.542
PI	0.61	0.547	0.239	0.815

*BMI* Body mass index, *AMH* AntiMullerian hormone, *FSH* Follicular stimulating hormone, *VI* Vascularization index, *FI* Flow index, *RI* Resistive index, *PI* Pulsatility index

^a^Using bootstrap resampling (times = 500)

## Discussion

Embryo freeze–thaw technology allows patients to choose the most appropriate time for transplantation, especially when the fresh transplant cycles are not available for some reason. Ultrasound-based imaging techniques can reflect multiple physiological and pathological conditions of the uterus. Personalized predictive tools that are rigorously developed and validated can be used in infertility patients to help more patients build healthy families [13]. In this community-based cohort study, we developed a quantifiable and simple nomogram to predict the clinical pregnancy in FET patients under the age of 40 years. After an internal validation, high stability of predictive accuracy was found in both training and validation cohorts. Decision curve analysis also demonstrated the clinical value of this nomogram. We also estimated the optimal cutoff values of each related factor for predicting the clinical pregnancy in FET cycles. Moreover, ultrasound and clinical baseline parameters are readily obtained in this model, so the nomogram has considerable practical value.

Different prediction models for the outcome of assisted reproductive technology on the basis of demographic information and clinical measurements have been developed in our country and abroad. The pregnancy capacity of patients receiving assisted reproduction treatment is relatively low, and the AUC of prediction models in reproductive medicine is ≤ 0.68 [14], and there are relatively few studies on the transfer cycle of freeze–thaw embryos. As early as 2008, Verberg et al. [15] established a Logistic regression model to predict the sustained pregnancy rate of women younger than 38 years of age after IVF mildly stimulated single embryo transfer. The analysis results showed that the body mass index, total gonadotropin dose required and the number of oocytes obtained were negatively correlated, while the availability of high-quality embryos was positively correlated with continued pregnancy. The predictive model provides an evidence-based guidance strategy for clinical diagnosis, but the predictive model is only used for a small group of women younger than 38 years old. In 2016, Mclernon et al. [16] established a logistic regression prediction model to estimate the live birth rate of multiple complete cycles of IVF treatment. This study can achieve customized prediction and estimation of the cumulative live birth rate of patients and help patients develop personalized treatment plans. In 2020, Jiang et al. [17] constructed a Logistic regression model based on IVF-ET and ICSI to predict clinical pregnancy outcome, and established a predictive model for various factors related to clinical pregnancy to diagnose the pregnancy success rate of patients, and provided relevant experiments for clinical practice. The area under the ROC curve is 0.669. Shui etc. [18] developed and validated a nomogram prediction model with its value of area under the receiver operating curve up to 0.949 for predicting pregnancy by using age and ultrasonographic factors including uterine peristalsis, uterine spiral artery, and ultrasound elastographic features. The sensitivity was 0.83 and specificity was 0.96. However, the research objects included were infertile people, not those who received assisted reproduction, and the number of subjects included was small, only 152 cases.

At present, many evaluations of COS assisted reproductive technology are still a non-consensus predictor, and there is no statistical explanation for the results obtained in clinical trials or for the same subject and different parts. The analysis of a model by using all related factors (i.e., full model) showed that including BMI, AMH, duration of infertility, endometrial pattern and VI did not improve prediction. The MFP model showed slightly better accuracy than the stepwise model, but the complicated formula restricted its use. (Details are described in the Supplemental Appendix). Therefore, the step-wise model is the simplest model under the premise of guaranteeing accuracy. The parameters included in this nomogram for FET cycles were female age, BMI, type of embryos transferred, the number of embryos transferred, endometrial thickness, the number of sub-endometrial blood, FI, RI, and PI.

A large number of studies have shown that age is an independent risk factor affecting pregnancy outcome of assisted reproductive technology [19]. There are two main factors affecting ART pregnancy outcomes in females: decreased ovum quality and impaired endometrial receptivity, which may be associated with increased chromosomal abnormalities and increased endometrial collagen content and decreased hormone receptors with age [20].

In vivo, most viable tissue, including the endometrium, requires an adequate blood supply and angiogenesis to develop and proliferate. A good blood supply to the endometrium/subendometrium is essential for a successful pregnancy and is supposed to play an important role in implantation and growth of the embryo. Therefore, blood supply to the base of the endometrium also affects endometrial receptivity. The uterine spiral artery is rich in blood vessels, which can directly reflect the blood perfusion of the microenvironment of the embryo implantation site, and the uterine spiral artery blood supply plays an important role in the endometrial receptivity [21]. If the hormone lacks periodic changes, it may cause the endometrial blood flow to become slender and the blood flow resistance will increase. PI and RI will increase sharply, resulting in poor blood perfusion and often obstructed blood supply [22]. However, the existence of blood supply disorder will cause slow endometrial proliferation and decreased uterine receptivity, which cannot provide strong conditions for embryo implantation. Therefore, low PI and RI are more conducive to embryo implantation. Some studies documented that relatively low RI and PI of endometrial vessels were correlated to achieving successful pregnancy, and RI was statistically different between patients in the pregnant group and those in the nonpregnant group [18]. However, other studies presented that there was no statistical correlation between these parameters of endometrial vessels and prediction of pregnancy results [23]. As mentioned above, the utility of endometrial and uterine vascularity measurement by transvaginal sonography (TVS) in predicting pregnancy outcome in the ET cycle had controversial results [24].

Uterine perfusion and fetal growth were different between the fresh and FET cycles. Cavoretto et al. [25] pointed out that IVF/ICSI pregnancies that had frozen blastocyst transfer (BT) demonstrated significantly better uterine perfusion and fetal growth compared to those that underwent fresh BT. In particular, uterine artery pulsatility index (UtA-PI) was as 14% lower in IVF/ICSI pregnancies conceived with frozen BT as compared to those from fresh BT. UtA-PI declines progressively throughout pregnancy, from 11 weeks gestation to term. A recent study [26] found that IVF/ICSI conceptions with thawed as opposed to fresh BT present a lower mean UtA-PI from 6 to 37 weeks, with greater fetal growth and a lower risk of small-for-gestational age (SGA). The study outlined the degree of uterine perfusion in pregnancy as a factor related to growth differences between fresh and thawed BT pregnancies. It has been shown that thawed BT after IVF/ICSI conceptions present greater CRLs compared with fresh, and both IVF/ICSI groups show smaller CRLs than the general population at 6–14 week. And this effect may favor birth weight difference of thawed versus fresh BT pregnancies. A meta-analysis of 11 studies and two large cohort studies [27, 28] found a reduced risk of SGA and fetal growth restriction by about 45% and an increased risk of being large-for-gestational age (LGA) by 30–90%, in IVF/ICSI pregnancies conceived after frozen BT, as compared to after fresh embryo transfer. Roy et al.[29] also found that the mean gestational age at birth and the mean birth weight of live births were significantly increased in neonates born in frozen cycles compared with fresh cycles.

The three-dimensional images obtained by three-dimensional ultrasound have higher clarity, which is conducive to the three-dimensional presentation of the spatial position relationship of the tissue structure [30]. The blood vessels and blood flow of tissues in the area of interest can also be quantified and expressed by blood flow parameters, which has great advantages in displaying the low-speed blood flow and blood flow parameters of the tiny tortuous uterine spiral. VI is the ratio of the number of color voxels to the total number of voxels in ROI, representing blood vessels in tissues. FI is the ratio of the total color intensity to the number of color voxels in ROI, which represents the blood flow intensity during 3D scanning. Several studies have suggested that endometrial vasculature indices in a cycle are associated with endometrial receptivity [31] and the following pregnancy rates [32]. However, there is also a 30-min vaginal ultrasonography study on frozen-thawed embryo transfer cards from Austria [33], suggesting that there is a significant difference in the volume of the endometrium between the pregnant group and the non-pregnant group. As the volume increased, the pregnancy rate increased, and there was no difference in the vascularization parameters FI, VFI, VI of the endometrium and subendometrium. Some researchers also believe that FI can objectively reflect the microvascular perfusion of the uterine spiral artery compared with VI, which simply reflects the blood vessels in the tissue, and is basically not affected by factors such as ultrasound sensitivity. Therefore, it has a higher value in predicting pregnancy outcome. There have been a meta-analysis indicated that the endometrial VI, FI, and VFI and subendometrial FI significantly differed between pregnant and nonpregnant women [34]. It probably owes to the fact that that there is no uniform standard of the location and range of sub-endometrium and endometrium [35, 36] And the difference in size and location of the sub-endometrium may cause different blood flow parameters. Kupesic et al. also demonstrated that subendometrial FI was significantly higher in pregnant cycles [37]. This study also reached the same conclusion, and FI was included in the model.

Patients with a standard BMI have a higher rate of clinical pregnancy [38] and a lower incidence of complications during pregnancy, which is conducive to live birth outcomes. Moreover, bFSH reflects the ovarian reserve; higher bFSH suggests poorer ovarian reserves and lower fertility [39].

Besides, we estimated the optimal cutoff values of each related factor to predict clinical pregnancy incidence, which may provide references in defining the best thresholds of female age, BMI, AMH, baseline FSH, endometrial thickness, VI, FI, RI, and PI for FET cycles.

The advantage of our study is that the traditional Logistic regression prediction model can provide the odds ratio and Cox regression risk ratio, and explain the statistical relationship between independent variables and dependent variables. Statistical power with each individual variable. In this study, according to the characteristics of the FET cycle, common clinical data and common ultrasound parameters were used to construct a prediction model suitable for the population receiving assisted reproductive technology in our center.

There are some limitations in this study. First, while the embryos used in the current study were of good morphological quality, the unknown genetic makeup of the embryos remains a problem. Second, the study was a single-center retrospective cohort study. Furthermore, the ages of these patients were younger than 40 years, and the relationship between the various factors of FET clinical pregnancy outcomes in older patients was not revealed. Finally, considering the many external influences during pregnancy, the node of the study only studied clinical pregnancy, and did not predict the birth rate.

## Conclusion

Our study not only develops a prediction model, but also an approach to building a prediction model that can be easily replicated with using basic clinical data and easily accessible ultrasound measurement indicators. Personalized quantitative prognostics convey an important message to patients that ART success is largely predictable, based on science and evidence [40]. This can minimize patients’ uncertainty and confusion and enhance their confidence in infertility treatment options. In addition, in order to better evaluate the role of endometrial receptivity in embryos with high implantation potential, it may be a future research direction to study the influencing factors of pregnancy outcome in PGT population alone.

## Supplementary Information

**Additional file 1: Table S1.** Prediction Performance of Three Prediction Models for Estimating the clinical pregnancy in FET cycles. **Fig. S1.** ROC curve of each related factors for clinical pregnancy in FET cycles.

## Data Availability

The datasets used and/or analyzed during the current study are available from the corresponding author on reasonable request.

## Abbreviations

IVF-ET: In vitro fertilization and embryo transfer
RIF: Recurrent implantation failure
BMI: Body mass index
VI: Vascularization index
FI: Flow index
RI: Resistive index
PI: Pulsatility index
LH: Luteinizing hormone
PRF: Pulse repetition frequency
hCG: Human chorionic gonadotropin
ROC: Receiver operating characteristic
AUC: Area under the ROC curve
PPV: Positive predictive value
NPV: Negative predictive value
PLR: Positive likelihood ratio
NLR: Negative likelihood ratio
MFP: Multivariable fractional polynomial
TVS: Transvaginal sonography
