# Supplementary material for: Nomogram prediction for the prediction of clinical pregnancy in Freeze-thawed Embryo Transfer
Source: BMC Pregnancy Childbirth. 2022 Aug 8;22:629. doi: 10.1186/s12884-022-04958-8 (PMC9361510; doi:10.1186/s12884-022-04958-8)

Supplemental Appendix

Table. S1 Prediction Performance of Three Prediction Models for Estimating the clinical pregnancy in FET cycles.

|  | MFP model | | Stepwise model | | Full model | |
| --- | --- | --- | --- | --- | --- | --- |
|  | Training cohort | Validation cohort | Training cohort | Validation cohort | Training  cohort | Validation cohort |
| AUC  (95% CI) | 0.706  (0.682, 0.731) | 0.692  (0.648, 0.736) | 0.698  (0.673, 0.723) | 0.699  (0.656, 0.743) | 0.698  (0.6732, 0.723) | 0.699  (0.656, 0.743) |
| Cut-off value | 0.6801 | 0.5059 | 0.6274 | 0.4856 | 0.6274 | 0.4856 |
| Specificity, % | 0.7420 | 0.4061 | 0.6254 | 0.3843 | 0.6254 | 0.3843 |
| Sensitivity, % | 0.5536 | 0.8773 | 0.6607 | 0.9147 | 0.6607 | 0.9147 |
| PPV, % | 2.1454 | 1.4773 | 1.7635 | 1.4855 | 1.7635 | 1.4855 |
| NPV, % | 0.6017 | 0.3021 | 0.5426 | 0.2221 | 0.5426 | 0.2221 |
| PLR | 0.7849 | 0.7075 | 0.7500 | 0.7087 | 0.7500 | 0.7087 |
| NLR | 0.4942 | 0.6691 | 0.5200 | 0.7333 | 0.5200 | 0.7333 |

**MFP model:**

1.49310+1.37192*(No. of Sub-endometrial blood=2) +2.14247*(No. of Sub-endometrial blood=3) +0.71160*(Type of embryos transferred=2)-6.71507*I(RI^1)-0.44554*I(Endometrial thickness ^-2)+0.50479*(No. of embryos transferred=2)-0.02370*I((FI/100)^-2)-4.45527*I(Age/100)^1)+0.35551*I((BMI10)^1)+2.21978*I(PI^2) -2.42731*I(PI^2 * log(PI))

**Full model:**

-1.89157-0.04625*Age+0.03622*BMI+0.69745*(Type of embryos transferred=2) +0.50034* ( No. of embryos transferred=2) +0.95587*(Endometrial thickness)+1.46040*(No. of Sub-endometrial blood=2) +2.24071*(No. of Sub-endometrial blood=3) +0.03437*FI -4.00098*RI+0.95536*PI

**Stepwise model:**

-1.89157-0.04625*Age+0.03622*BMI+0.69745*(Type of embryos transferred=2) +0.50034* ( No. of embryos transferred=2) +0.95587*(Endometrial thickness)+1.46040*(No. of Sub-endometrial blood=2) +2.24071*(No. of Sub-endometrial blood=3) +0.03437*FI -4.00098*RI+0.95536*PI

AUC: area under curve.

*****Using Bootstrap resampling (times = 500)

Fig. S1 ROC curve of each related factors for clinical pregnancy in FET cycles


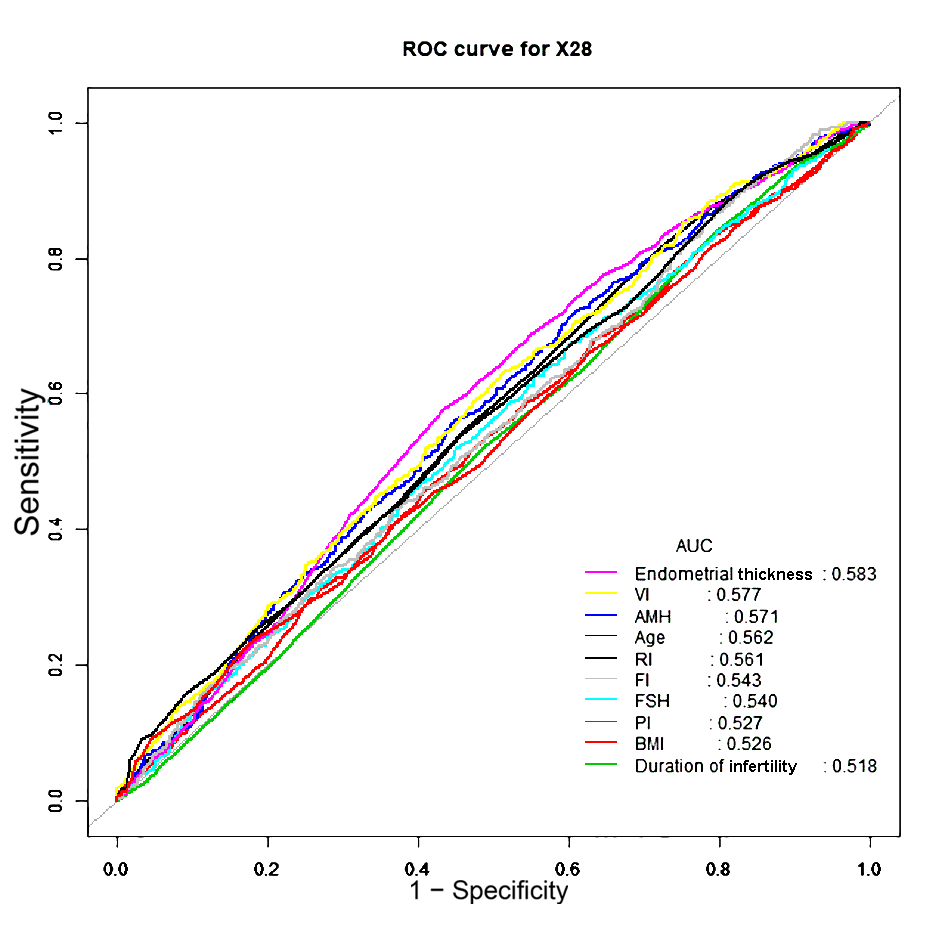

Supplement: Supplementary file 1 — Additional file 1: Table S1. Prediction Performance of Three Prediction Models for Estimating the clinical pregnancy in FET cycles. Fig. S1. ROC curve of each related factors for clinical pregnancy in FET cycles. [file 12884_2022_4958_MOESM1_ESM.docx]
